# Hygiene practices and factors influencing intestinal parasites among food handlers in the province of Belgarn, Saudi Arabia

**DOI:** 10.7717/peerj.14700

**Published:** 2023-01-20

**Authors:** Abdulrahman S. Alqarni, Majed H. Wakid, Hattan S. Gattan

**Affiliations:** 1 Sabt Al-Alaya General Hospital, Directorate of Health Affairs, Ministry of Health, Bisha, Saudi Arabia; 2 Faculty of Applied Medical Sciences, Medical Laboratory Technology, King Abdulaziz University, Jeddah, Saudi Arabia; 3 Special Infectious Agents Unit, King Fahd Medical Research Center, King Abdulaziz University, Jeddah, Saudi Arabia

**Keywords:** Hygiene practices, Intestinal parasites, Food handlers, Socio-demographic status, Risk factors, Belgarn, Saudi Arabia

## Abstract

**Background:**

This study aimed to evaluate the association between socio-demographic status, and hygienic habits among food handlers with intestinal parasitic infections.

**Methods:**

It was a cross-sectional study in which 112 participants were recruited, from Belgarn province of Saudi Arabia. The descriptive analysis was used to evaluate demographic data and categorical variables. The association between sociodemographic characteristics and Relative Risk regression analysis was performed for each investigated factor. *p*-value of <0.05, was assumed to be statistically significant.

**Results:**

One hundred and twelve food handlers with a mean age of 33.5 ± 9.2 years were included in this study. The food handlers were from 15 different countries (11 Asians and four Africans). The majority of the food handlers were cooks (87, 77.7%), and waiters and dish washers (24, 21.4%). Among them, 106 (94.6%) used uniforms, and gloves at work. In hand washing practices, 104 (92.9%) wash their hands with soap before handling and preparing food and eight (7.1%) wash without soap, 98 (87.5%) wash with soap before meals and 14 (12.5%) wash without soap, 105 (93.8%) wash with soap after visiting toilets and seven (6.2%) wash without soap. Twenty-five (42.3%) of infected food handlers are not used to trim their fingernails. Intestinal infection was observed in 59 (52.68%) participants with mean age (32.5 ± 8.1 years) for infected participants.

**Conclusion:**

In this study, food handlers had a high prevalence of intestinal parasites. Although some of the food handlers had a high level of education, the infection with intestinal parasites was detected. In addition to regular screening for intestinal parasites among food handlers, there is a need for educational programs on proper hygiene habits, modes of transmission and prevention of the infection.

## Introduction

Intestinal parasitic infections (IPIs) are among the common infectious diseases worldwide, mainly in developing countries (Workineh, Almaw & Eyayu, 2022). Intestinal parasites that inhabit gastrointestinal system cause several clinical pathological aspects, such as children growth delay, poor school scores, malnutrition, anemia, and cancer (Torgerson et al., 2015; Wakid, 2020). According to the World Health Organization, 24% of the world population are likely to be infected with soil-transmitted helminths, whereas over three billion are infected with intestinal parasites but have no clinical symptoms (WHO, 2020). In endemic countries, IPIs can cause severe morbidity and mortality (Ünal et al., 2020).

Helminth and protozoa parasitic infection can occur through different routes. In an infected person with helminths, infective stages develop into adult worms in the intestinal tract, which then lay eggs (Hotez et al., 2006). Intestinal protozoa spread easily through fecal-oral route of contaminated food or water with the infective stages (Centers for Disease Control and Prevention (CDC), 2019; Haghighi et al., 2009; WHO, 2015). The high rates of IPIs reflect the poor hygienic behavior in the society. Climate conditions, inadequate sanitation, economic issues, a lack of access to safe drinking water, poor nutrition, and cultural norms are all linked to IPIs (Opara et al., 2012). Individuals are more vulnerable to IPIs than others, including those who reside in or travel to tropical or subtropical locations, immune impaired patients, youngsters, and the elderly (El-Sherbini & Abosdera, 2013).

When sanitation is not practiced, the food handlers may transmit intestinal protozoa and some helminth directly by the fecal-oral route without the need for intermediate hosts (Duboscq, Romano & MacIntosh, 2019).

Based on previous studies conducted among non-Saudi residents in Bahrah, Kingdom of Saudi Arabia, 22.8% of infected participants were food handlers. IPIs have been linked to personal cleaning practices, drinking water sources, and housing style (Wakid, 2020). In another study, Taha, Soliman & Banjar (2013) investigated foreign laborers in Al-Madina Al-Munawarah. Food handlers had the second highest infection rate (18.8%). In Makkah, Zaglool et al. (2011) determined that 23% of the food handlers at the nutrition department of Alnoor Specialist Hospital are infected with intestinal parasites. In 2006, Wakid conducted a study in Jeddah and reported that 50.15% of the investigated food handlers were infected with intestinal parasites (Wakid, 2006).

In Ethiopia, Yeshanew, Tadege & Abamecha (2021), reported IPIs among 44.6% of food handlers with one or more intestinal parasites. The main associated factors were poor hand washing practices, older age, untrimmed fingernails, insufficient training, and a lack of frequent health checkups.

The community’s prevalence of intestinal parasites has a detrimental influence on socioeconomic position (Hussain et al., 2019). Developmental disturbances, vitamin A insufficiency, iron deficiency anemia, and weak educational achievement are the consequences of IPIs on children (Crompton & Nesheim, 2002). In addition, IPIs in immune compromised patients, such as those with HIV, transplant patients and patients with renal dialysis, may lead to serious complications (Omrani et al., 2015).

Awareness regarding intestinal infection among food handlers is needed. To effectively reduce IPIs in endemic regions, comprehensive control measures involving better sanitation, intestinal infection awareness, and chemotherapy are required (WHO, 2013).

In our previous study in the province of Belgarn among food handlers, we investigated the prevalence, types of intestinal parasites and compared between the performance of the techniques (Alqarni, Wakid & Gattan, 2022). The current study was carried out to evaluate the hygiene practices and factors influencing the distribution of intestinal parasites among food handlers in the province of Belgarn, Saudi Arabia.

## Materials & Methods

This cross-sectional study in Belgarn province of Saudi Arabia was conducted according to the guidelines of the Declaration of Helsinki and approved by the research and ethics committee of the Faculty of Applied Medical Sciences at King Abdulaziz University, Jeddah, Saudi Arabia (Approval number FAMS-EC2021-08), with direct supervision from the Municipality of Belgarn province.

Belgarn is a small province, located on mountaintops in the southwestern region of Saudi Arabia. All 112 food handlers working in this province were involved in the current study between April and September 2021, without any exclusion criteria, and each participant signed an informed consent form. The infected food handlers were contacted to visit Sabt Al-Alaya General Hospital for treatment and follow-up.

A questionnaire was used to capture socio-demographic information and hygiene related question including wearing of gloves/gown, washing habits as a lifestyle, nail trimming practices and source of drinking water. In addition to variables related to awareness about intestinal parasites. Face-to-face interviews were conducted with participants to fill the questionnaire.

This study performed variant laboratory testing on the collected stool samples including macroscopic examination, direct wet smears, sedimentation formal ether technique, permanent staining with trichrome and modified Kinyoun’s, rapid immunochromatographic diagnostic tests, and real-time PCR. The procedures were conducted as described in detail in our previous study (Alqarni, Wakid & Gattan, 2022).

The data collected was entered and analyzed using the Statistical Package for the Social Sciences (SPSS version 25; IBM, Inc., Chicago, IL, USA). The descriptive analysis was used to evaluate demographic data and categorical variables. Association between socio-demographic characteristics and IPIs was analyzed using the chi-square (X^2^). Relative Risk (RR) regression analysis was performed for each investigated factor. *p*-value of <0.05, was assumed to be statistically significant.

## Results

A total of 112 food handlers participated in the study with age ranging between 20 and 65 years (mean ± SD = 33.5  ± 9.2) years. 76.7% were aged below 40 years and were all males.

Out of 112, 73 (65.2%) participants were married. Majority of them belonged to education level up to high school (48, 42.85%).

The majority of the food handlers were cookers, (87, 77.7%), waiters and dish washers (24, 21.4%), and serving both (1, 0.9%). Most of them (73, 65.1%) were working in restaurants, or in cafeterias (20, 17.9%), in confectionery shops (6, 5.3%) or in pizza shops (5, 4.5%). The majority of food handlers 106 (94.6%) used uniforms, and gloves at work.

The food handlers were from 15 different countries: Bangladesh (24, 21.4%), Egypt (16, 14.3%), India (14, 12.5%), Yemen (12, 10.7%), Pakistan (10, 8.9%), Afghanistan (9, 8.0%), Syria (7, 6.2%), Nepal (6, 5.4%), Turkey (4, 3.6%), Burma (4, 3.6%), Sudan (2, 1.8%), and (1, 0.9%) from Tunisia, Morocco, Jordan and Indonesia.

Intestinal infection was observed in 59 (52.68%) participants. Information on the species-specific prevalence of intestinal parasitic infection and the comparative analysis of the detection techniques were presented in the previous study (Alqarni, Wakid & Gattan, 2022).

Table 1 illustrates the distribution of infection according to age group of food handlers. The majority (83%) were young workers between 20 and 39 years. Participants who were unaware about the mode of infection/prevention of intestinal parasites had higher percentages of parasitic infection, however there was no statistically significant difference (*p* > 0.05) between infected and uninfected subjects.

**Table 1:** Association between intestinal parasitic infection and socio demographic characteristics and awareness among infected and non-infected participants. This table illustrates the distribution of infection according to age group, education level, service they provide, accommodation type and period since last vacation to his country.

Variables	Categories	Infected *n* = 59 (%)	Uninfected *n* = 53 (%)	*P*-value
Age group (years)	20–29	26 (44.06)	19 (35.84)	>0.05
	30–39	23 (38.98)	18 (3.96)	
	40–49	7 (11.86)	11 (20.75)	
	50–60	3 (5.08)	4 (7.54)	
	>60	0	1 (1.88)	
Education level	Illiterate	9 (15.25)	4 (7.54)	0.05
	Primary School	12 (20.33)	17 (32.07)	
	Middle School	5 (8.47)	7 (13.20)	
	High School	24 (40.67)	24 (45.28)	
	Bachelor	9 (15.25)	1 (1.88)	
Awareness about intestinal parasites	Aware	10 (16.94)	17 (32.07)	>0.05
	Not aware	44 (74.57)	35 (66.03)	
	Not sure	5 (8.47)	1 (1.88)	
Service they provide	Cooks	45 (76.27)	42 (79.24)	>0.05
	Waiters	13 (22.03)	11 (20.75)	
	Dish washer/serving	1 (1.6)	0	
Accommodation type	Alone	5 (8.47)	5 (9.43)	>0.05
	With family	4 (6.77)	2 (3.77)	
	With other workers	50 (84.74)	46 (86.79)	
Period since last vacation to his country	<6 months	14 (23.72)	3 (5.66)	>0.05
	6–11 months	3 (5.08)	4 (7.54)	
	1–2 years	17 (28.81)	17 (32.07)	
	>2 years	25 (42.37)	29 (54.71)	

Participant’s infection rates in relation to their nationalities are shown in Table 2. The mean age for infected participants was 32.5 ± 8.1 years, while 34.5 ± 10.1 years for uninfected participants with no significant difference (*p* > 0.05).

**Table 2:** Infection distribution among nationalities. This table shows in detail the number of the infected and uninfected food handlers from each nationality.

**Nationality**	Total (*n*)	Uninfected (*n*)	Infected (*n*)	**Infection rate (%)**	***p*** **-value**
Bangladeshi	24	17	7	29.17	>0.05
Egyptian	16	6	10	62.50	
Indian	14	6	8	57.14	
Yemeni	12	3	9	75.00	
Pakistani	10	2	8	80.00	
Afghan	9	4	5	55.56	
Syrian	7	4	3	42.86	
Nepalese	6	3	3	50.00	
Turkish	4	2	2	50.00	
Burmese	4	4	0	0.00	
Sudanese	2	1	1	50.00	
Jordanian	1	0	1	100.00	
Tunisian	1	0	1	100.00	
Indonesian	1	1	0	0.00	
Moroccan	1	0	1	100.00	
**Total**	112	53	59	52.68	

Based on level of education, intestinal parasites were identified most frequently in food handlers with high school education (24, 40.7%), with primary school education (12, 20.3%), illiterate and bachelor holders, each (9, 15.3%), and among middle school educational attainment (5, 8.4%).

Infection rates were higher among food handlers who lived with other workers than among those who lived alone or with their families, but without significant difference (*p* > 0.05). Similarly, those who recently arrived to Saudi Arabia revealed higher infection rate, but there was no significant difference.

In this study, drinking water source was not statistically significant factor for IPIs (*p* > 0.05), as shown in Table 3. According to the state of fingernails trimming, 25 (42.3%) of infected food handlers are not used to trim their fingernails, no statistically significant difference (*p* > 0.05) was observed between infected and uninfected subjects.

**Table 3:** Frequency of hygiene habits among infected and uninfected participants. This table shows that participants who were unaware about the mode of infection/prevention of intestinal parasites had higher percentages of parasitic infection, however there was no statistically significant difference (*P* > 0.05) between infected and uninfected subjects.

***Practice***	Infected (59) *n* (%)	Uninfected (53) *n* (%)	*p*-value
**Uniform** **/** **Gloves usage**			
No	1 (1.69)	5 (9.43)	>0.05
Yes	58 (98.3)	48 (90.56)	
**Washing hands before handling/preparing food**				
Yes, with soap	54 (91.52)	50 (94.39)	
Yes, without soap	5 (8.47)	3 (5.66)	
**Washing hands before meals**				
Yes, with soap	51 (86.44)	47 (88.67)	
Yes, without soap	8 (13.55)	6 (11.33)	
**Washing hands after visiting toilet**				
Yes, with soap	54 (91.52)	51 (96.22)	
Yes, without soap	5 (8.47)	2 (3.77)	
**Frequency of shower (weekly)**				
1	0	1 (1.88)	
2–3	3 (5.08)	3 (5.66)	
>4	56 (94.91)	49 (92.45)	
**Trimmed nails**				
No	25 (42.37)	29 (54.71)	
Yes	34 (57.62)	24 (45.28)	
**Drinking water source**				
Bottled	30 (50.84)	26 (49.05)	
Filtered	29 (49.15)	27 (50.94)	

**Table 4:** Regression analysis for prediction of infected persons. This table shows that drinking water source, fingernails trimming and washing hands habits were not statistically significant factor for IPIs.

**Variables**	***p*-value**	**RR (95% CI)**
Illiterate or primary school (versus higher education)	0.660	0.898 (0.556–1.451)
Cooker versus washer or waiters	0.059	1.728 (0.979–3.051)
Accommodation Type with family or other workers (versus alone)	0.859	1.077 (0.477–2.430)
Period since last vacation to his country >2 years	0.192	0.733 (0.460–1.169)
Un awareness about intestinal parasites	0.095	1.607 (0.921–2.802)
Uniform/gown/gloves usage	0.080	2.962 (0.877–10.006)
Washing hands before handling/preparing food without soap (versus with soap)	0.563	1.311 (0.524–3.278)
Washing hands before meals without soap (versus with soap)	0.720	1.138 (0.562–2.303)
Washing hands after visiting toilet without soap (versus with soap)	0.305	1.699 (0.617–4.681)
Frequency of shower <4	0.592	0.768 (0.293–2.015)
Trimmed nails	0.192	1.364 (0.855–2.176)
Drinking bottled water (versus filtered)	0.850	1.046 (0.657–1.664)

**Notes.**

RR relative risk CI confidence interval

As shown in Table 4, regression analysis was conducted for prediction of infected persons using education, responsibility, accommodation type, period since last vacation to his country, awareness, protective clothes, washing hands, shower frequency, trimming nails and drinking water source as covariates. None was considered independent predictor for infection with intestinal parasites.

## Discussion

The current study was carried out in Belgarn province to evaluate hygiene practices and influencing factors of intestinal parasites among food handlers in the province of Belgarn, Saudi Arabia. In this study, intestinal parasites were found in 52.7% of the 112 food handlers. In a recently published article, we presented the details of the prevalence, types of detected intestinal parasites and comparison between the performed techniques (Alqarni, Wakid & Gattan, 2022).

Previous studies on food handlers in Saudi Arabia revealed varying rates of IPIs prevalence, ranging from 23% to 50.15% (Wakid, 2006; Zaglool et al., 2011; Taha, Soliman & Banjar, 2013; Wakid, 2020). All these previous studies showed that most of food handlers were Asians between the ages of 20 and 40, which is consistent with the findings of our study.

The present study reported a high rate of parasitic infection among participants from different nationalities. It is important to emphasize that most of food handlers in Saudi Arabia are from countries with high infection rates.

The rate of infection was observed to be influenced by educational level, with statistically significance (*p* = 0.05). This is consistent with findings from two studies conducted in Qatar and Iran, which found that educational level had a significant impact on the prevalence of infections (Balarak et al., 2016; Younes et al., 2021). It could be concluded that as level of educational attainment improves, the awareness about parasitic infections will do so. The results of the logistic regression were not significant.

Other socio demographic factors such as age and marital status were not found to be predictive of infection. Hand washing practices were evaluated and found to be good. 91.5% of food handlers claimed that they wash their hands with soap before handling and preparing food, as well as after using the toilet, while 8.5% wash without soap. Other studies have reported significant results for the same predictors, therefore this finding seemed to be inconsistent with our findings (Alemu et al., 2019; Wakid, 2020). According to a previous study, hand washing decreased IPIs up to 68% by avoiding ingestion of the infective stage of the parasite (Mahmud et al., 2015).

Our present study investigated awareness about intestinal parasites in relation to length of residency for food handlers in Saudi Arabia. 48 food handlers had less than 5 years of residency in Saudi Arabia, and 23% of them are aware about intestinal parasites. On the other hand, 64 food handlers spent more than 5 years of residency in Saudi Arabia, and almost 35% claimed they know about intestinal parasites. These data emphasizes the importance of pre-employment screening and health education to improve social habits and food safety, particularly for new food handlers. There was an irregularity in the conduct of strict hygiene and an absence of appropriate monitoring because there was no significant difference between having a health card and the infection rate.

There was no statistically significant correlation between having trimmed fingernails and the incidence of intestinal parasites (*p* > 0.05). This contrasts with the results of a study in Ethiopia that found a significant association with infection (Alemu et al., 2019). Untrimmed fingernails may contain infectious agents that cause disease or serve as a good route of autoinfection.

Surveillance systems should encourage prevention and control activities in order to minimize the prevalence of these diseases, eliminate the incidence and ultimately improve public health. In addition, prevention and control practices will also lead to overall economic growth. Thus, precise surveillance is essential for the control of parasitic diseases. These strategies are becoming more possible than before, due to the discovery of effective drugs, the development and simplification of certain diagnostic methods and the progress of parasite population biology (Bahk et al., 2018). Integrated control measures require to be implemented to efficiently control IPIs, including improved hygiene, education programs, and chemotherapy (Nyantekyi et al., 2014).

## Conclusions

In this study, food handlers had a high prevalence (52.68%) of intestinal parasites. In addition, most of the recently returned food handlers from their countries after vacation were infected. Although some of the food handlers had a high level of education, the infection with intestinal parasites was detected. Therefore, not only screening is required, but there is also a need for additional education on proper hygiene habits and disease transmission. Continuous epidemiological surveillance through regular surveys is required to address the incidence of IPIs. Municipalities should undertake interventions such as training of all food handler on food safety, particularly hand washing mostly all the time, as well as nail clipping. Educational programs are important to raise food handlers’ awareness of these infections and the risks they pose to themselves and their customers, which may contribute to infection transmission reduction.

##  Supplemental Information

10.7717/peerj.14700/supp-1 Supplemental Information 1 All results related to the hygiene practices and factors influencing intestinal parasites among food handlers in the province of Belgarn, Saudi Arabia

10.7717/peerj.14700/supp-2 Supplemental Information 2 Questionnaire form of the study this is the questionnaire form used in the study of Intestinal parasitic Infection among food handlers in Belgarn Province, sausdi arabia.

## Additional Information and Declarations

### Data Availability

The following information was supplied regarding data availability: The raw data is available in the Supplemental Files.
