# Supplementary material for: Hygiene practices and factors influencing intestinal parasites among food handlers in the province of Belgarn, Saudi Arabia
Source: PeerJ. 2023 Jan 20;11:e14700. doi: 10.7717/peerj.14700 (PMC9869772; doi:10.7717/peerj.14700)
Supplement: Supplemental Information 2 — this is the questionnaire form used in the study of Intestinal parasitic Infection among food handlers in Belgarn Province, sausdi arabia. [file peerj-11-14700-s002.pdf]

إستمارة إستبيان ـ العدوى الطفيلية المعوية بين العاملين في مجال الغذاء في محافظة بلقرن

Intestinal Parasitic Infection among Food Handlers in Belgarn Province -Questionnaire Form

Serial #: الرقم التسلسلي:

Shop name: إسم المحل:

Contact No.: رقم الجوال:

Name:
Gender: 1) Male 2) Female
Age (years): 3) 20-29 4) 30-39 5) 40-49 6) 50-60
Nationality: 7) Indian 8) Bangladeshi 9) Saudi 10) Yemeni 11) Egyptian 12) Sudanese 13) Syrian 14) Afghan 15) Pakistani 16) Turkish 17) Others ……………..
Weight (kg):
Height (cm):
Marital status: 18) Single 19) Married 20) Others …………….
Educational status: 21) Illiterate 22) Primary School 23) Middle School 24) High School 25) Bach. or higher
Responsibility: 26) Cooker 27) Waiter and/dish washer 28) Serving both
Monthly income (SAR): 29) < 1000 30) 1000-1999 31) 2000-2999 32) >3000
Establishment type: 33) Restaurant 34) Cafeteria 35) Pizza shop 36) Confectionery 37) Coffee shop 38) Bakery 39) Ice cream and juice shop
Years of residency in Saudi Arabia: 40) < 1 41) 1-5 42) 6-10 43) > 10
The period of stay from the last vacation: 44) < 6 months 45) 6 - < 12 months 46) 1-2 years 47) > 2 years
Accommodation type: 48) With other workers 49) With family 50) Alone
Drinking water source: 51) Bottled 52) Filtered 53) Tap 54) Wells
Awareness about intestinal parasites: 55) Aware 56) Not aware 57) Not sure
Complain from abdominal symptoms: 58) Yes 59) No
Uniform/gown usage/gloves: 60) Yes 61) No
Washing hands before handling and preparing food: 62) Yes, with soap 63) Yes, without soap 64) Rarely 65) No
Washing hands before meals: 66) Yes, with soap 67) Yes, without soap 68) Rarely 69) No
Washing hands after using toilet: 70) Yes, with soap 71) Yes, without soap 72) Rarely 73) No
Frequency of having shower: 74) Once per week 75) 2-3 times per week 76) > 4 times per week
Having trimmed fingernails: 77) Yes 78) No
Validity of health card: 79) Valid 80) Expired

More information:
